# Traumatic Native Hip Dislocations: An Audit at a Major Trauma Centre and Assessment of Clinical Practice at Centres Across the United Kingdom

**DOI:** 10.7759/cureus.58314

**Published:** 2024-04-15

**Authors:** Zayd Jawad, Wahid Abdul, Jonathan Topping, James Dunn, James Lewis, Khitish Mohanty

**Affiliations:** 1 Trauma & Orthopaedics, Morriston Hospital, Swansea, GBR; 2 Trauma & Orthopaedics, University Hospital of Wales, Cardiff, GBR; 3 Orthopaedics, University Hospital of Wales, Cardiff, GBR; 4 Accident and Emergency, University Hospital of Wales, Cardiff, GBR

**Keywords:** major trauma centre, orthopaedics trauma, retrospective audit, survey study, hip avascular necrosis, polytrauma, traumatic hip dislocation, native hip dislocation

## Abstract

Introduction: Native hip dislocations are defined as traumatic dislocations of the hip, typically high-energy and associated with polytrauma. The majority of these injuries occur following motor vehicle accidents (MVAs). Due to the inherent stability of the hip joint, a significant force is required to cause dislocation. It is critical that such injuries are managed and reduced in a timely manner. We evaluated the current practice in a major trauma centre (MTC) in Cardiff and gathered information from emergency departments (EDs) in Wales and MTCs around the United Kingdom (UK).

Methods: We did an evaluation of the current practice with a retrospective audit of all traumatic native hip dislocations presenting to the MTC at Cardiff from August 2018 to February 2021. Data was obtained from Trauma Audit and Research Network (TARN), medical records, radiology and theatre management systems. An online survey was developed and disseminated to EDs in Wales and MTCs across the UK.

Results: There were 15 traumatic hip dislocation cases over the period evaluated. Sixty percent of cases were due to MVA. Eighty-six percent of patients had an associated fracture, with one Pipkin type IV fracture dislocation. The mean time to reduction from injury was 532 minutes (240-804 minutes), with 28.6% reduced within 6 hours and 71.4% reduced within 12 hours. Two patients had reduction performed in the ED (mean time to reduction, 275 minutes). There was one occurrence of avascular necrosis (AVN) and one of chondrolysis at the follow-up. The response rate to the survey was 80% and 83% in Wales and MTCs nationally, respectively. The majority (82%) of departments did not have an established pathway in place for managing traumatic native hip dislocations with a preference for reduction in the operating theatre.

Conclusion: Native hip dislocations are rare, high-energy injuries associated with significant morbidity. The available evidence suggests time to reduction is imperative in reducing the risk of future complications. The establishment of a pathway to guide management and having a mechanism to perform reductions in the ED may produce significant reductions in this time, impacting outcomes.

## Introduction

Native hip dislocations are high energy traumatic events associated with polytrauma. Previous research has suggested that the complications associated with these injuries such as avascular necrosis (AVN) is influenced by the timing of reduction. [1]. The hip is an inherently stable joint requiring significant force to dislocate it. This stability is due to the osseous anatomy and the surrounding ligamentous structures and musculature [2]. Thus, hip dislocations are rare events with up to 95% occurring in motor vehicle accidents (MVAs) [3,4].

Due to the high-energy trauma often involved in these injuries, there is an association with concomitant injuries including fractures to the pelvis and femoral head, fractures at other sites and visceral injuries requiring management in an intensive care setting. A systematic assessment using advanced trauma life support (ATLS) principles is critical to ensuring that the patient is thoroughly assessed and resuscitated. The development of central major trauma centres (MTCs) has further refined the management of polytrauma patients with multiple specialists in a single site able to address each critical injury in one place, avoiding transfers and unnecessary delays to critical care [5].

The first and only MTC in Wales opened in September 2020. We evaluated the management of native hip dislocations before and after this was established. The audit aimed to answer multiple questions regarding native hip dislocations including the following: (1) Are native hip dislocations being reduced in a timely manner (within 6 and within 12 hours)? [1,6]. (2) Where are these reductions being performed and who are the team members involved? (3) How does this practice compare with emergency departments (EDs) across Wales and MTCs across the United Kingdom?

With the results of this evaluation, we aimed to arrange and introduce a protocol to standardise management of native hip dislocations with the ultimate aim of more reductions being performed in the ED setting.

## Materials and methods

A retrospective audit was performed of all traumatic native hip dislocations presenting to the MTC in South Wales from August 2018 to February 2021. The MTC was established in September 2020 and as such data would include presentations both before and after the introduction of the MTC. The audit was registered and approved with the local audit department. There were no standards to audit against. Instead, the practice was measured against a 6-hour and a 12-hour window, using a previous research for reference [1,6].

Data was obtained from the Trauma Audit and Research Network (TARN), medical notes, Welsh Ambulance Service (WAS) documentation and call log, local departmental theatre management and radiology systems as well as Welsh Clinical Portal (WCP). The timing of injury was noted from the moment the WAS logged the call to an incident. An online survey (Table 1) was drafted, revised and submitted to the Welsh ED and UK MTC Clinical Leads (CLs).

**Table 1:** Survey questions disseminated to EDs in Wales and to MTCs across the United Kingdom ED, emergency department; MTC, major trauma centre

Question
Does the ED at your hospital have a pathway in managing native traumatic hip dislocations?
In your hospital, where are native traumatic hip dislocations reduced?
What type of native hip dislocations can be reduced in the ED?
Which speciality is responsible for performing the closed reduction in the ED?
What sedation do you use for closed reduction of native hip dislocations in the ED?

## Results

Fifteen traumatic native hip dislocations presented between August 2018 and February 2021, with five presenting during the establishment of the MTC (Table 2). All 15 cases were posterior dislocations. The mean age at presentation was 36 years (4-74 years, interquartile range, or IQR, 22 years) with 93% (n=14) of cases being over the age of 18 years. Eighty percent (n=12) were male and 60% (n= 9) were due to MVAs. Approximately 86% of patients had an associated fracture with their dislocation, with all of these patients having an acetabular fracture and a single incidence of a Pipkin IV combined femoral head and acetabular fracture. Almost half of the patients (47%) were polytrauma.

**Table 2:** Demographics of patients presenting with native hip dislocations MVA, motor vehicle accident; IQR, interquartile range

Demographics		Result, n	Result, %
Number of dislocations		15	
Gender	Male	12	80
	Female	3	20
Age		36 years (4-74 years, IQR 22)	
Paediatric (<18 years)		1	6
Mechanism	MVA	9	60
	Sports	3	20
	Pedestrian vs. car	1	6
	Crush injury	1	6
	Fall	1	6
Associated fracture	Acetabulum	12	80
	Combined femoral head and acetabulum	1	6
Polytrauma	Isolated	8	53
	Polytrauma	7	47
Intubated pre-admission		2	13

Details regarding times, location and assessments are displayed in Table 3. The time of injury has been measured as the time of call to the WAS, which is logged and noted electronically. Of note, 66% of patients were seen immediately upon arrival in the ED by the Trauma and Orthopaedics team as part of a pre-alerted trauma call. The mean time to review by the Orthopaedics team was 34 minutes over (0-157 minutes, IQR 80 minutes). In 79% (n=11) of patients, hip dislocations were reduced within 12 hours of injury, while in 29% (n=4) of patients were reduced within 6 hours of injury with a mean time of 532 minutes overall (240-804 minutes). Of the 15 patients, two arrived intubated and had their reductions performed in the ED (mean time to reduction, 275 minutes), with the rest transferred to the theatre for reduction under anaesthesia with a mean time to reduction of 576 minutes. One patient self-presented, and an accurate measure of the time of injury could not be made. All patients had a documented neurovascular assessment before and after the intervention except where patients were intubated and ventilated at the time of presentation. Every patient had a post-operative neurovascular assessment documented while 87% of patients received a post-reduction computed tomography (CT) scan.

**Table 3:** Results of the retrospective audit of management of native hip dislocations before and after the establishment of the MTC MTC, major trauma centre; AVN, avascular necrosis; DVT, deep vein thrombosis; IQR, interquartile range

Data collected			Result, n	Result, %
Location of reduction		ED resuscitation	2	13
		Theatre	13	87
Time to the orthopaedic review (minutes)			34 (0-157, IQR 80)	
Immediate orthopaedic review			10	
Mean time to reduction from injury (minutes)		Overall	532 (240-804, IQR 421)	
		Theatre	576 (240-804, IQR 293)	
		ED	275 (260-290, IQR 30)	
Time to reduction	<6 hours		4	29
	<12 hours		11	79
MTC patients			5	33
Mean time to the orthopaedic review (minutes)			26 (0-132, IQR 66)	
Mean time to reduction from injury (minutes)			574 (276-798, IQR 435)	
Neurovascular status documentation		Pre-reduction	13	87
		Post-reduction	15	100
Intubated prior to arrival			2	13
Neurovascular injury		Pre-reduction	2	13
		Post-reduction	1	6
Post-reduction CT scan			13/15	87
Complications		AVN	1	6
		Chondrolysis	1	6
		DVT	1	6

At the follow-up, there was one occurrence of chondrolysis confirmed on MRI, where reduction occurred 710 minutes after the injury. During a two-year follow-up period, the patient underwent a repeat MRI scan and arthroscopy, and was subsequently discharged following physiotherapy. There was also one case of avascular necrosis confirmed on imaging at the 16-month follow-up; the patient subsequently underwent total hip replacement, and active follow-up is ongoing. There was one complication of deep vein thrombosis (DVT) occurring three months following open reduction internal fixation (ORIF) of the acetabular fracture.

The online survey was conducted on SurveyMonkey (San Mateo, CA) and the form included the questions displayed in Table 1. The results of these surveys are displayed in Tables 4-5. There was an 83% response rate from Welsh EDs. Twenty percent of those that responded had an established pathway for managing native hip dislocations. Seventy percent of respondents stated reductions were to take place in an operating theatre, with the remaining 30% allowing reduction in the ED provided no associated fracture was found.

**Table 4:** Results of surveys sent to EDs across Wales ED, emergency department

Data collected		Result, n	Result, %
Surveys sent		12	
Responses		10	83
Established pathway for a native hip dislocation		2	20
Location of reduction	ED	3	30
	Theatre	7	70
Sedating agent		Fentanyl and propofol combination	

**Table 5:** Results of surveys sent to MTCs across the United Kingdom MTC, major trauma centre; ED, emergency department

Data collected		Result, n	Result, %
Surveys sent		27	
Responses		22	81.5
Established pathway for a native hip dislocation		5	23
Location of reduction	ED	9	41
	Theatre	12	59
Sedating agent		Fentanyl and propofol combination	

Nationally, there was an an 81.5% response rate from MTCs. Twenty-three percent of MTCs had an established pathway in place. Approximately 59% of respondents stated reductions took place in an operating theatre with the remaining 41% performing reductions in the ED (77% of this cohort performed reductions in the ED even in the presence of associated fractures). Across Wales and MTCs nationally, the common anaesthetic agent of choice was a combination of propofol and fentanyl.

## Discussion

Native hip dislocations are rare injuries involving high-energy trauma and are clinical emergencies where outcomes are associated with timely reduction. Currently, the leading cause of these injuries is MVAs, accounting for up to 95% events [4,7,8]. This study involved posterior dislocations, which account for 90% of native hip dislocations [9]. Because of the high-energy nature of these injuries, patients often arrive with multiple concomitant injuries with haemodynamic instability requiring attention in other areas before addressing the hip. Indeed, the dislocated hip may not be apparent until imaging is obtained [9]. In terms of the short- and long-term effects, they are associated with complications including sciatic nerve injury, AVN, chondrolysis and post-traumatic osteoarthritis [10-14].

An audit of our clinical practice demonstrates the difficulty in reducing these injuries in a timely manner, particularly when transferred to the theatre. In our series, the majority of patients had their hip dislocation reduced more than six hours later from the time of injury, while both patients who had reduction in the ED were managed successfully within six hours. Three patients breached the 12-hour time to reduction. Of note, the two patients who developed chondrolysis and AVN did so having been reduced within a 12-hour timeframe.

Due to the dynamic and complex nature of such situations, a transfer from the site of injury to the MTC may present challenges, requiring time to stabilise the patient and involving large teams on scene as well as two paramedics and, if intubated, an airway-trained clinician to safely transport the patient to the appropriate centre [15]. A significant period of time is often elapsed by the time of presentation to the ED, with a priority to stabilising and resuscitating the patient via a primary survey, a necessity prior to addressing the injury. Once addressed, and with the patient stabilised, the consensus regarding hip dislocations is that reduction with minimal delay is vital to patient outcomes [1,16-18].

Given the rarity of these injuries, there have been few studies involving large numbers of patients, and variable outcomes have been reported. In a study of 98 adults, Hougaard and Thomsen found a more than 10-fold increase in the rate of AVN when dislocated hips were reduced more than six hours later from the time of injury (4.8% to 52.9%) [1]. Indeed, such was the significance of time to reduction that the only other influencing factor found was severity of dislocation, although the number of patients with Pipkin grade IV (dislocation with fracture of the head or neck) dislocations was small when compared with grades I and II [19].

Sahin et al. in their long-term follow-up study of 62 patients used a 12-hour window as a benchmark [18]. They found a secondary osteoarthritis rate of 16% occurring between 2 and 10 years of follow-up. They also noted an AVN rate of 8%. They concluded that patients had a better prognosis if reduction was performed within 12 hours but did not show significance in regard to AVN rates and time to reduction.

Kellam and Ostrum performed a systematic review and meta-analysis and found a highly variable prognosis in the 13 studies they included [6]. They noted that all studies were retrospective cohort studies. They ultimately found an odds ratios (OR) of 5.62 of developing AVN when such reductions occurred more than 12 hours after injury, although only two studies included observed time to reduction and AVN rates [6,18,20].

Our results showd a significant increase in time to reduction in patients who received reduction in the operating theatre against those who received reduction directly in the ED. The majority of patients in our centre were reduced in a theatre setting, with the two cases managed in the ED already being intubated. As a result of the transfer to the theatre, patients were delayed a further 300 minutes (five hours) on average, significantly increasing the risk of complications. There are multiple factors involved in this delay, including availability of staff to safely transfer, prior stabilisation in the ED, arrangement of imaging in the theatre, availability of an emergency theatre and anaesthetic time prior to starting. Dislocations of hips in the ED are often reduced in a theatre setting due to the potential forces required against the musculature to relocate the joint [21]. In contrast with prosthetic hip joint dislocations, native hips are thought to be more difficult to provide sufficient relaxation to overcome these forces [22]. A 10-year review by Bressan et al. in 2014 suggested such reductions in the ED were safe in the paediatric population [23]. Conscious sedation has therefore been proposed as a method to facilitate reduction in the ED [21]. As part of the primary survey, patients undergo a polytrauma CT scan unless critically unstable. As such, an associated fracture can be diagnosed or excluded, and a prompt decision can be made about the suitability of attempted reduction in the ED. This is dependent on the availability of staff able to safely sedate the patient in that environment. From our survey, the anaesthetic agents of choice for sedation in the ED were found to be propofol and fentanyl, which has been shown to be a safe combination of drugs that take effect and are eliminated quickly [24].

Many methods have been described for reducing both posterior and anterior hip dislocations [25]. The technique of choice is up to that of the clinician, in their experience. Post-reduction imaging is essential for further evaluation. Radiographs are a quick and inexpensive method for the confirmation of reduction [26]. CT scans are the imaging modality of choice to exclude previous unidentified fractures or loose bodies within the joint space [9]. Furthermore, close follow-up is advised to assess the hip joint and monitor for complications such as AVN and chondrolysis. The imaging modality of choice for the detection of these complications is MRI [27].

There are also certain limitations to our study. As stated previously, across two and a half years, 15 hip dislocations were seen, reflecting the rarity of these events, although projected to become more common with the advent of the major trauma centre and the complex polytrauma scenarios that it attracts. The small sample size therefore means we are unable to attain sufficient power with regard to the significance of delays in reduction and outcomes. However, our results are in line with previous evidence [1,18]. To further build an evidence base, we propose ongoing prospective data collection via TARN. Follow- ups are also limited by timeframes, with complications potentially appearing much further in the timeline of events, or patients being potentially lost to follow-up if they are repatriated closer to home.

**Figure 1:**
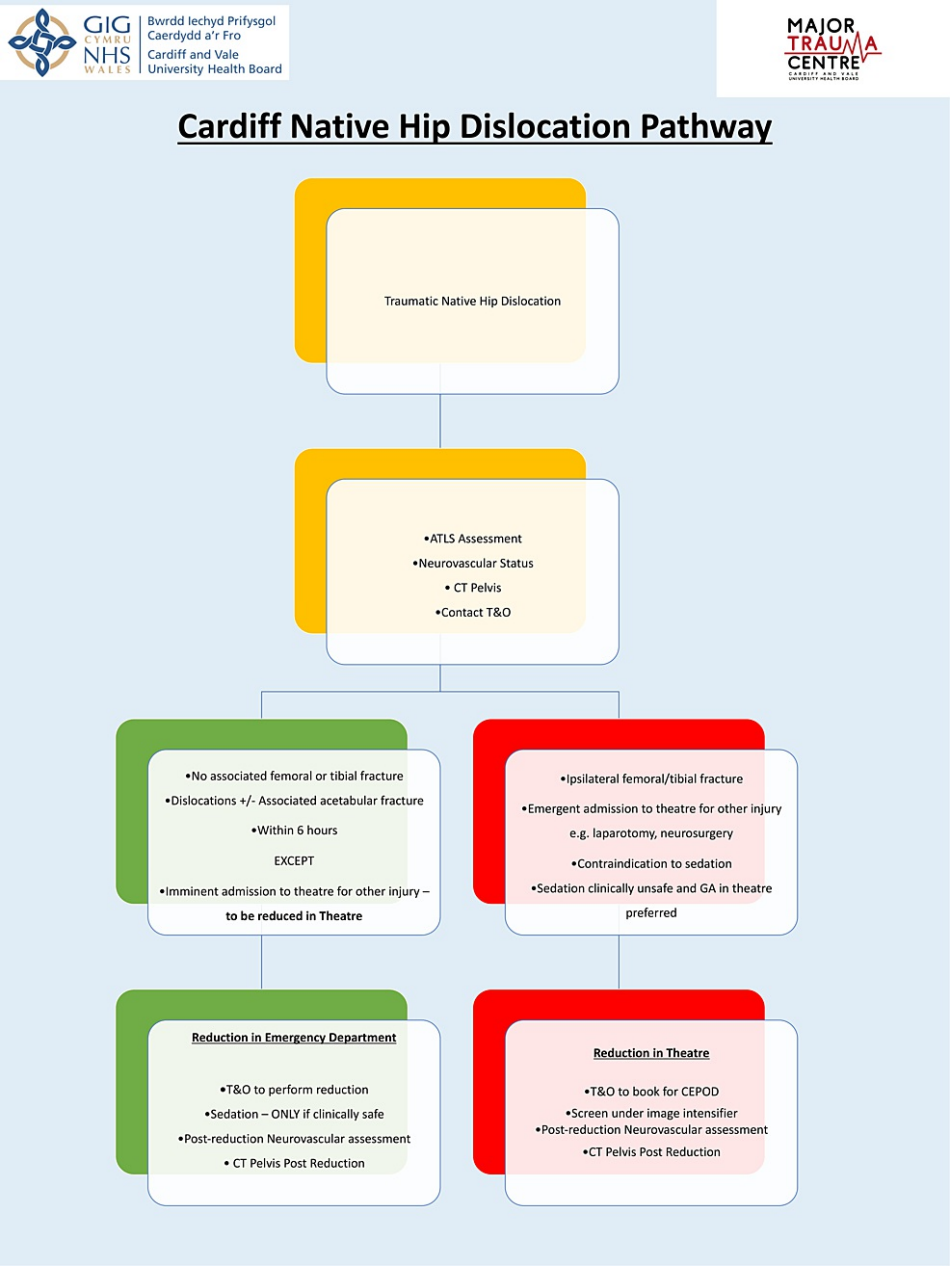
Native hip dislocation pathway introduced in February 2023 T&O, trauma and orthopaedics; MTC, major trauma centre; ATLS, advanced trauma life support; GA, general anaesthetic; CEPOD, Confidential Enquiry into Perioperative Deaths (a term used for emergency theatre) Figure presented is a creation of the authors, now in use in the MTC.

From this study, we identified that time to transfer and subsequent time to theatre were significant hurdles in reducing native hip dislocations in a timely manner. With large teams present in the ED and staff from both anaesthetics and EDs capable of administering safe sedation, we propose reducing such injuries directly in the ED in conjunction with the trauma and orthopaedics team. Provided there is no associated fracture, many hours could be saved, reducing the risk of future complications. With that, we have developed a pathway that was introduced in 2023 to guide the management of traumatic hip dislocations in the ED (Figure 1).

## Conclusions

Native hip dislocations are potentially life-changing injuries with permanent consequences if not managed in a timely manner. With the development of the MTC, patients with complex, high-energy trauma are presenting to single central units and being managed effectively with improved outcomes in morbidity and mortality. We have demonstrated that patients having dislocations reduced in the theatre risk significant delays compared to those who are managed in the ED. With trauma team availability, rapid attainment of imaging and the ability to administer sedation and anaesthetic agents safely in the ED, we propose reduction of simple native hip dislocations in the ED as a significant time-saving, outcome-improving treatment method. A pathway has subsequently been introduced to guide the management of such cases in future, which has been the result of a multi-disciplinary approach and agreement. Ongoing prospective data collection will guide management and help to monitor outcomes in the future.

## References

[1] Hougaard K, Thomsen PB (1986). Traumatic posterior dislocation of the hip—prognostic factors influencing the incidence of avascular necrosis of the femoral head. Arch Orthop Trauma Surg (1978).

[2] Tsutsumi M, Nimura A, Akita K (2022). Clinical anatomy of the musculoskeletal system in the hip region. Anat Sci Int.

[3] Alshammari A, Alanazi B, Almogbil I, Alfayez SM (2018). Asymmetric bilateral traumatic hip dislocation: a case report. Ann Med Surg (Lond).

[4] Weber CD, Lefering R, Sellei RM, Horst K, Migliorini F, Hildebrand F, TraumaRegister Dgu (2022). Traumatic hip dislocations in major trauma patients: epidemiology, injury mechanisms, and concomitant injuries. J Clin Med.

[5] Moran CG, Lecky F, Bouamra O (2018). Changing the system - major trauma patients and their outcomes in the NHS (England) 2008-17. EClinicalMedicine.

[6] Kellam P, Ostrum RF (2016). Systematic review and meta-analysis of avascular necrosis and posttraumatic arthritis after traumatic hip dislocation. J Orthop Trauma.

[7] Epstein HC (1973). Traumatic dislocations of the hip. Clin Orthop Relat Res.

[8] Rosenthal RE, Coker WL (1979). Posterior fracture-dislocation of the hip: an epidemiologic review. J Trauma.

[9] Dawson-Amoah K, Raszewski J, Duplantier N, Waddell BS (2018). Dislocation of the hip: a review of types, causes, and treatment. Ochsner J.

[10] Brandão GF, Américo LR, Soares CB, Faria RG, Teixeira LE (2010). Traumatic posterior dislocation of the hip in children: report on five cases. Rev Bras Ortop.

[11] Dreinhöfer KE, Schwarzkopf SR, Haas NP, Tscherne H (1994). Isolated traumatic dislocation of the hip. Long-term results in 50 patients. J Bone Joint Surg Br.

[12] Hillyard RF, Fox J (2003). Sciatic nerve injuries associated with traumatic posterior hip dislocations. Am J Emerg Med.

[13] Hougaard K, Thomsen PB (1987). Coxarthrosis following traumatic posterior dislocation of the hip. J Bone Joint Surg Am.

[14] Upadhyay SS, Moulton A, Srikrishnamurthy K (1983). An analysis of the late effects of traumatic posterior dislocation of the hip without fractures. J Bone Joint Surg Br.

[15] Berkeveld E, Popal Z, Schober P, Zuidema WP, Bloemers FW, Giannakopoulos GF (2021). Prehospital time and mortality in polytrauma patients: a retrospective analysis. BMC Emerg Med.

[16] Epstein HC (1974). Posterior fracture-dislocations of the hip; long-term follow-up. J Bone Joint Surg Am.

[17] Pietrafesa CA, Hoffman JR (1983). Traumatic dislocation of the hip. JAMA.

[18] Sahin V, Karakaş ES, Aksu S, Atlihan D, Turk CY, Halici M (2003). Traumatic dislocation and fracture-dislocation of the hip: a long-term follow-up study. J Trauma.

[19] Romeo NM, Firoozabadi R (2018). Classifications in brief: the Pipkin classification of femoral head fractures. Clin Orthop Relat Res.

[20] Brav EA (1962). Traumatic dislocation of the hip: army experience and results over a twelve-year period. JBJS.

[21] Dursteler BB, Wightman JM (2000). Etomidate-facilitated hip reduction in the emergency department. Acad Emerg Med.

[22] Frymann SJ, Cumberbatch GL, Stearman AS (2005). Reduction of dislocated hip prosthesis in the emergency department using conscious sedation: a prospective study. Emerg Med J.

[23] Bressan S, Steiner IP, Shavit I (2014). Emergency department diagnosis and treatment of traumatic hip dislocations in children under the age of 7 years: a 10-year review. Emerg Med J.

[24] Swanson ER, Seaberg DC, Mathias S (1996). The use of propofol for sedation in the emergency department. Acad Emerg Med.

[25] Waddell BS, Mohamed S, Glomset JT, Meyer MS (2016). A detailed review of hip reduction maneuvers: a focus on physician safety and introduction of the Waddell technique. Orthop Rev (Pavia).

[26] Adams J, Koerner M, Williams C, Tanner SL, Sridhar MS, Schaller TM, Jeray KJ (2020). The dislocated hip on CT scan: an argument for the initial pelvic radiograph in trauma patients. J Surg Orthop Adv.

[27] Amarnath C, Muthaiyan P, Mary TH, Mohanan S, Gopinathan K (2018). Idiopathic chondrolysis of hip in children: new proposal and implication for radiological staging. Indian J Radiol Imaging.

